# Complex Membrane Channel Blockade: A Unifying Hypothesis for the Prodromal and Acute Neuropsychiatric Sequelae Resulting from Exposure to the Antimalarial Drug Mefloquine

**DOI:** 10.1155/2015/368064

**Published:** 2015-10-20

**Authors:** Jane C. Quinn

**Affiliations:** Plant and Animal Toxicology Group, School of Animal and Veterinary Sciences, Graham Centre for Agricultural Innovation, Charles Sturt University, Boorooma Street, Wagga Wagga, NSW 2650, Australia

## Abstract

The alkaloid toxin quinine and its derivative compounds have been used for many centuries as effective medications for the prevention and treatment of malaria. More recently, synthetic derivatives, such as the quinoline derivative mefloquine (bis(trifluoromethyl)-(2-piperidyl)-4-quinolinemethanol), have been widely used to combat disease caused by chloroquine-resistant strains of the malaria parasite, *Plasmodium falciparum*. However, the parent compound quinine, as well as its more recent counterparts, suffers from an incidence of adverse neuropsychiatric side effects ranging from mild mood disturbances and anxiety to hallucinations, seizures, and psychosis. This review considers how the pharmacology, cellular neurobiology, and membrane channel kinetics of mefloquine could lead to the significant and sometimes life-threatening neurotoxicity associated with mefloquine exposure. A key role for mefloquine blockade of ATP-sensitive potassium channels and connexins in the substantia nigra is considered as a unifying hypothesis for the pathogenesis of severe neuropsychiatric events after mefloquine exposure in humans.

## 1. Background

Quinine is an alkaloid toxin found in the bark of the South American cinchona (quina-quina) tree [1]. Quinine and its derivative compounds have been used since the 1800s as a homeopathic remedy for analgesia and also as an effective treatment for malaria [2]. Despite its proven efficacy as a schizonticidal agent against the malaria parasite* Plasmodium falciparum* during its intraerythrocytic phase, quinine suffers from a number of contraindications which have made it problematic as an effective therapeutic including a low therapeutic index and high incidence of adverse side effects [3]. Despite this, and a lack of efficacious alternatives, quinine remained the most widely used antimalarial until the 1920s when a new breed of compounds was discovered. Drugs such as chloroquine then became the treatment of choice for malarial prophylaxis and were used widely in all areas with endemic malaria for many decades [4].

By the 1950s, increasing levels of chloroquine resistance necessitated a push for the discovery of novel compounds for malarial chemoprophylaxis [5]. The synthetic quinoline derivative mefloquine (bis(trifluoromethyl)-(2-piperidyl)-4-quinolinemethanol) [6], an effective antimalarial but potent neurotoxin, was identified as part of this discovery process. First synthesised in the late 1960s, mefloquine's potent antimalarial properties were identified as part of a two-phase US military drug discovery programme that was mounted to identify novel antimalarial compounds for use primarily in their theatres of operation in Southeast Asia [4, 5, 7–10]. Studies showed that chloroquine and mefloquine acted via the same erythrocyte accumulation mechanism, but with mefloquine showing greater affinity, likely the mechanism for its increased efficacy both as a treatment and a prophylactic compared to chloroquine [11]. Despite historical knowledge of quinine and quinoline-induced related adverse drug reactions [7, 12], including hearing loss, visual disturbances, and severe hypoglycaemia [13–15], mefloquine was expeditiously developed with the assistance of the US Government and the pharmaceutical company Hoffmann La Roche [16, 17] and released following limited clinical testing [18, 19].

Over the next twenty years mefloquine was widely advocated as the drug of choice for travellers to areas known to be endemic for chloroquine-resistant malaria [20] such as sub-Saharan Africa [21, 22]. During this time, it was reported to be “well tolerated, safe, and effective” [23] despite coincident reports of significant neuropsychiatric side effects in isolated cases [24]. During the 1990s and 2000s, an increasing body of clinical case material reported significant neuropsychiatric side effects presenting in patients taking mefloquine for malarial prophylaxis [25–33]. Clinical presentation included a range of neurological symptoms in previously healthy individuals which included tremor, balance disturbances, fatigue, nausea, dizziness, anxiety or panic attacks, sleep disturbances including insomnia and vivid nightmares, visual disturbances, and hearing loss [31, 34], as well as severe neuropsychiatric sequelae including major personality change, psychosis, seizures, suicidal ideation, and suicide completion [26, 27, 31–33, 35, 36].

This “toxidrome,” a collection of significant neurological symptoms affecting balance, vision, hearing, memory, personality, and emotional status, has now been described as a limbic encephalopathy with central vestibulopathy [37], an overarching diagnosis covering all the possible manifestations of this complex neurotoxicity. This review will consider how mefloquine might induce this wide range of clinical effects in the central nervous system and explores current knowledge surrounding its binding partners at the cell surface. It will also present evidence suggesting destabilisation or destruction of the brain's central pacemaker, the substantia nigra, as a unifying hypothesis underlying many of the neuropsychiatric features of mefloquine toxicity.

## 2. Pharmacokinetics and Bioavailability: Implications for Clinical Presentation of Neurotoxicity Resulting from Mefloquine Exposure

The incidence of adverse reactions to mefloquine treatment and/or prophylaxis has long been a point of controversy. Early studies suggested that patients did not experience the very severe neuropsychiatric side effects that had been reported with chloroquine [38–42] but as increasing numbers of adverse events began to be reported in the literature, this opinion changed. Recently, controlled clinical trials have suggested that the incidence of neuropsychiatric side effects in travellers using mefloquine for malarial prophylaxis as well as those for treatment of malaria was more than a hundredfold greater than had been suggested in early studies investigating drug safety [32, 43–46]. However, despite significant reporting of the clinical manifestations of mefloquine toxicity [31], factors underlying the variability in presentation and severity of clinical signs observed in a subset of patients presenting with significant adverse reactions have yet to be fully elucidated.

Some of the pharmacological properties of mefloquine, which contribute to its efficacy as an antimalarial, may also contribute to its neurotoxicity. Mefloquine has a long plasma half-life (13–28 days), which contributes to its efficacy as a prophylactic treatment achievable by easy weekly dosing [47, 48]. Mefloquine is also highly lipophilic and exhibits stereoselective passage across the blood brain barrier (BBB) [48–51]. In the brain, highest concentrations have been reported in the hippocampus and subcortical areas in rodent studies [52, 53] with samples from human postmortem tissues shown to be up to 10-fold higher than plasma levels [50, 54].

One mechanism likely to cause increased retention of mefloquine in the CNS is via inhibition of the membrane efflux pump P-glycoprotein. P-gp (also known as ATP-Binding Cassette protein 1, ABC1), encoded by the Multi-Drug Resistance gene 1 (MDR1), is a transmembrane protein found lining the brain capillary endothelium that plays a specific role in central neuroprotection by restricting access of lipophilic molecules across the BBB [55]. The normal function of P-gp is to protect the brain from neurotoxic attack by limiting CNS access to complex molecules; mefloquine has been shown to be a potent inhibitor of P-glycoprotein [56], blocking its action at the BBB and causing retention of mefloquine in nervous tissues.

Effectiveness of the CYP450 enzyme superfamily in oxidative enzymatic degradation of common pharmaceuticals, including mefloquine, is likely to also play a significant role in the presentation and severity of neuropsychiatric side effects as genetic polymorphisms in the enzymes CYP2D6, CYP2C19, CYP3A4, and CYP1A2 [57] have all been implicated in adverse reactions to common antidepressants including incidences involving significant violence and suicide [58]. In these cases, ultrarapid metabolism was linked to suicide and extreme violence [58, 59] resulting from rapid conversion to toxic metabolites or bioactive drug production, whilst activity caused by single or multiple allelic mutation can cause failure of systemic depletion of the parent compound, with increased risk of adverse reactions to common neuropsychiatric drugs [57]. Recently, the relationship between treatments responses and genetic polymorphism at the CYP2A6 and CYP2B6 loci were investigated in patients receiving dual artesunate-mefloquine treatment for* P*.* falciparum malaria* [60] where mutations in CYP2A6 were related to poorer treatment outcomes due to reduced metabolic conversion of artesunate to dihydroartemisinin. Serum level of mefloquine was not measured but it might be assumed that these would also be high as low dose chloroquine has been shown recently to induce severe neuropsychiatric symptoms in an individual with mutation of the CYP2A6 and altered CYP450 activity [61], symptoms that were further exacerbated by treatment with other common psychiatric drugs. These findings suggest that allelic variation in CYP450 significantly increases the risk of severe neuropsychiatric sequelae on exposure to quinoline derivatives and further pharmacogenetic studies are warranted to investigate this possibility.

Mutations in genes associated with cellular metabolism of toxic compounds are not the only polymorphisms implicated in increased risk of adverse neuropsychiatric events associated with quinoline therapy. Mutations at the MNDR1 locus have also been shown to be associated with a heightened sensitivity to mefloquine* in vitro* [62] as well as being correlated with an increased incidence of neuropsychiatric side effects in humans particularly in women [63]. The malarial homologue of P-gp is also implicated in conferring chloroquine resistance to some strains of* Plasmodium falciparum* [63, 64] suggesting a common role and important role for these transmembrane proteins in restricting passage of mefloquine into cells under normal condition.

The highly lipophilic nature of mefloquine is also likely to be a contributory factor to the variable presentation of adverse neuropsychiatric events in humans. It has been shown that travellers with a low body weight index, taking mefloquine for malarial prophylaxis, showed an increased likelihood of adverse reactions than those taking chloroquine or the combination therapy chloroquine and proguanil [32]. This is likely due to reduced binding of the active compound to body fat stores and therefore a preferential compartmentalisation in other lipophilic tissues, such as the brain. Certainly volume of distribution is known to play a key role in drug toxicity and it has been well documented for mefloquine's parent molecule quinine that compartmentalisation, elimination, and excretion are all affected by age and health status of the patient. In particular, rates of elimination have been found to be slower in the elderly and those suffering from acute malaria than young or well individuals [47, 65] and similar effects have been identified in patients with active malarial infection treated with mefloquine [66].

Together, these findings suggest that variability in plasma half-life, activity of efflux pumps of the CNS/vascular interface, and compartmentalisation, as well as underlying genetics regulating neuroactive drug sensitivity and metabolism, may all contribute to toxic loading of mefloquine in the CNS and therefore the highly variable presentation of adverse events in a subpopulation of mefloquine users. Thus, multiple factors affecting drug retention and concentration in the CNS make accurate prediction of adverse events in patients exposed to mefloquine highly problematic.

## 3. Mefloquine Receptor Channels and Binding Partners: Relating Clinical Signs of Mefloquine Neurotoxicity to Intracellular Interactions in the Central Nervous System

It has been well described that toxic loading of mefloquine in the CNS can be subject to significant interpersonal variation; however, this does not fully explain the highly varied expression of adverse side effects reported by individuals exposed to therapeutic levels of mefloquine. At toxic doses, mefloquine has been widely reported to cause neuronal dysfunction, axonal degeneration, and neuronal cell death in a variety of cell types of the central nervous system [67–70], yet in some patients it appears to be able to elicit these significant and detrimental effects at much lower doses. How mefloquine induces multiple effects across a range of different neuronal subtypes is not yet clear but understanding its membrane binding partners in the CNS, and effects on cell membrane excitability, could give some clues as to its varying modes of toxicity in the human brain.

## 4. Mefloquine Exerts Receptor Blockade of ATP-Sensitive Potassium Channels (**K**_ATP_)

One family of neuronal cell membrane channels likely to play a key role in mefloquine neurotoxicity are the octomeric ATP-sensitive potassium (K_ATP_) channels. K_ATP_ channels are found in a wide range of tissues, including cardiomyocytes, smooth muscle, hormone secreting cells of the pancreas, and neurons of the CNS [71, 72]. K_ATP_ channels contain two components, a pore-forming Kir subunit and a sulfonylurea receptor (SUR) subunit, members of the ATP-binding cassette superfamily [73, 74]. A number of variants of these receptor subcomponents have now been identified, with different subunit pairings found in different cell types in the body. The Kir subunit forms the channel pore and contains the ATP-inhibition site, whilst the SUR subunit is sensitive to sulphonylureas and channel agonists [75–77]. K_ATP_ channels have been shown to play a fundamental role in glucose homeostasis via their activity in insulin-secreting islet cells of the pancreas [78–81] and are also widely distributed throughout the brain, in particular, being found within the cell membranes of postsynaptic *γ*-amino-butyric acid (GABA) inhibitory neurons of the cerebral cortex, substantia nigra pars reticulata (SNr), pars compacta (SNc), and cerebellum [73, 77, 82–84].

**Figure 1:**
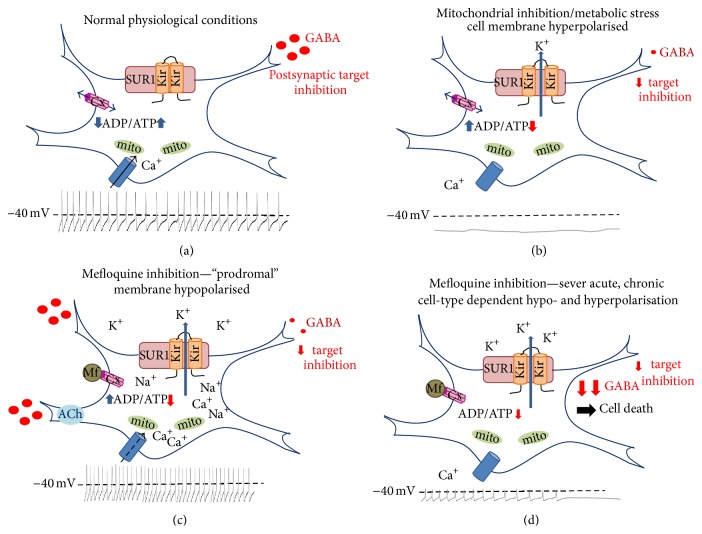
K_ATP_ channels found within the cell membrane of both pre- and postsynaptic neurons of the substantia nigra (SNr) and ventral tegumental area (VTA), as well as other brain regions. In the SNr and VTA, the majority of K_ATP_ neurons are dopaminergic and exert GABAergic inhibition on their postsynaptic targets. (a) Within these cells, under normal physiological conditions, K_ATP_ channels are closed and ligand-gated calcium channels are free to open allowing hypopolarisation of the cell membrane and tonic excitation. Connexin channels (Cx) are also open allowing for exchange of ions, metabolites, and second messengers to enable appropriate electrical coupling. (b) Under conditions of metabolic stress or hypoxia, ATP depletion causes K_ATP_ channels to open and Ca^+^ channels to close, hyperpolarising the cell membrane and conferring neuroprotective proprieties by inhibiting neuronal excitation. Inhibition of target cells is diminished due to reduction in GABA release, stimulating hypopolarisation in postsynaptic targets. Connexin channels are hypothesised to remain open under these conditions. (c) In the presence of low concentrations of mefloquine, K-ATP channels are inhibited and remain open in the absence of metabolic stress, causing potassium efflux from the cell and increased transport of sodium into the cell through this open cation pore. This sodium influx causes an initial hypopolarisation, increasing tonic firing and initially enhancing presynaptic GABA release. Increased activity of Na^+^ K^+^-ATPase transmembrane channels increases metabolic stress, ensuring that K_ATP_ channels remain open. GABA release is, initially, further enhanced by inhibition of cholinesterase (ChE) which results in accumulation of endogenous acetylcholine (ACh) in the presynaptic terminal. Continued exposure is likely to result in intracellular ATP depletion, and finally GABA depression, resulting in a loss of postsynaptic inhibition. In addition, mefloquine (Mf) blocks connexin channel transport, further dysregulating intra- and intercellular excitability, giving rise to additional neuropsychiatric symptoms such as focal cortical or limbic seizures. (d) Exposure to high levels of mefloquine, or continued long-term exposure, would result in complete inhibition of K_ATP_ channel closure, continued connexin channel blockade, and permanent dysregulation of postsynaptic inhibition by presynaptic GABAergic inhibition as well as exerting significant neuronal metabolic stress, finally resulting in metabolic cell death in the basal neuron and potentiating excitotoxic cell death target neurons in other brain regions. Adapted from Liss and Roeper [140].

In neurons, K_ATP_ channels modulate the availability of ATP for cellular metabolism by responding to depletion of ATP resources within the cell. Their activation results in opening of the K_ATP_ channel causing membrane hyperpolarisation. Therefore, in neurons under normal metabolic conditions, K_ATP_ channels are closed, only opening when the intracellular ATP/ADP ratio decreases sufficiently to required metabolic homeostasis to be restored (Figure 1). What is important in the context of neurotoxicity is that this modulation in ATP-dependent membrane excitability confers dual properties to the cell: (1) influencing maintenance of normal spontaneous firing patterns as well as (2) conferring neuroprotective properties to the cell in instances of metabolic stress, such as in hypoxia or ischaemia [82, 85]. K_ATP_ channel blockade alters neuronal excitability and negates the neuroprotective effect, potentially leaving cells susceptible to ischaemic or excitotoxic cell death under conditions of metabolic stress or disease (Figure 1).

Initial clues as to a role for K_ATP_ channels in mefloquine toxicity came from studies investigating metabolic abnormalities in patients treated with quinine and its derivatives. Mefloquine, chloroquine, quinoline, and quinine have all been shown to block the activity of K_ATP_ channels in islet cells of the pancreas, increasing insulin secretion [86, 87]. This finding, associated with reports of severe hypoglycaemia in patients undergoing treatment with both quinine and mefloquine for malarial prophylaxis or acute disease [15, 88, 89], suggests a similar mode of action for the two compounds in the pancreas in humans [86]. Quinine and quinoline derivatives, therefore, are likely to impede K_ATP_ channel function in other tissues such as the brain and there is growing evidence to support this hypothesis.

## 5. **K**_ATP_ Channel Blockade and Limbic Seizures

K_ATP_ are widely distributed throughout the brain and have been shown to be present in GABA-responsive neurons of the cerebral cortex, substantia nigra pars reticulata (SNr), pars compacta (SNc), and cerebellum [73, 82]. Of particular relevance to this review is the finding that GABAergic neurons of the SNr have been shown to express K_ATP_ channels, cells which are known to play a key role in maintenance of normal spontaneous firing activity in the brain [84, 90]. This spontaneous excitation pattern has been described as the “fast spiking pacemaker” of the central nervous system, instructing the tonic output activity of the basal ganglia and other subcortical regions, and is fundamentally required for normal neurological function [91].

Under normal metabolic conditions, K_ATP_ channels in the SNr are closed and cells exhibit a high level of spontaneous activity, suppressing seizure activity by release of GABA onto postsynaptic terminals (Figure 1(a)). Conversely, in states of metabolic tension, such as hypoxia, these channels are activated causing a protective hyperpolarisation due to calcium efflux, and thus reducing membrane excitability (Figure 1(b)) [82]. K_ATP_ channels in the SNr have been shown to play a significant role in both neuronal protection and seizure suppression [82, 92] with the majority of neurons in the SNr being GABAergic and exhibiting high levels of spontaneous firing [90]. Mefloquine inhibition of SNr K_ATP_ channels would open the membrane pore, initially maintaining high levels of spontaneous activity and GABA release regardless of metabolic status (Figure 1(c)). Continued inhibition would result in depletion of cellular metabolic stores, reducing GABA release [93, 94], and finally cell death of SNr neurons (Figure 1(d)). This blockade would also confer an inability to regulate excitability in target neurons of the mesostriatal dopaminergic pathway, similar to the hyperexcitability observed in hypoglycaemia [95], increasing dopamine release and potentially resulting in excitotoxic cell death in target neuron populations (Figure 1). K_ATP_ membrane mefloquine channel inhibition would also reduce the neuroprotective ability of midbrain neurons to respond to metabolic ATP depletion making them more sensitive to metabolic stress in states or injury or disease. Mefloquine could therefore induce both neuronal dysfunction and cell death in this critical regulatory region of the brain.

**Figure 2:**
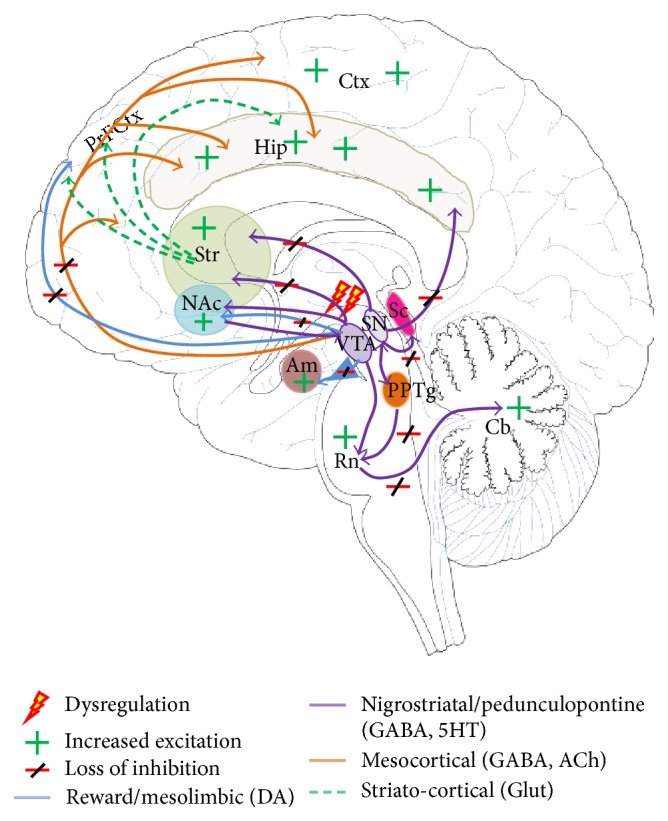
Pathways in the brain implicated in the clinical presentation of mefloquine toxicity. Loss of ascending inhibition within the nigrostriatal-pedunculopontine, mesolimbic and mesocortical pathways, resulting in hyperactivation of neurons in the amygdala, striatum, nucleus accumbens, and cortical and hippocampal areas, as well as within descending pathways to the Raphe nuclei, and cerebellum could give rise to the complex neuropsychiatric sequelae observed in patients exposed to prophylactic and treatment doses of mefloquine. Secondary disinhibition of striato-cortical pathways is implicated in seizure and motor and cognitive changes observed in affected individuals.

Another interesting and relevant finding is that extracellular dopamine levels in the striatum increases as the base firing rate of SNr neurons increases [96]. This biochemical change could potentially deregulate the delicate balance between serotonergic and dopaminergic control of the mesolimbic system (Figure 2), such as what is seen in the alterations of mood and behaviour associated with states of addiction to psychostimulants [97] and neuronal synchronisation characteristic of limbic seizures. Limbic seizures are classified as psychogenic seizure events without major epileptiform changes, which result in paroxysmal episodic alteration in cognitive function, behaviour, and emotional control [98–101]. These two conditions have apparent similarities to mefloquine toxicity. A number of studies support this hypothesis. Firstly, the SNr has been identified to be the site of action of the anticonvulsant topiramate in the intrahippocampal pilocarpine model of limbic seizures, exerting its action by either direct connection to the hippocampus or indirect subcortical connections via the striatum (Figure 2) [102–106]. Secondly, sustained opening of K_ATP_ channels was induced by mild hypoxia in mice without a functional Kir6.2 subunit, causing neuronal depolarisation and enhanced membrane sensitivity, sufficient to cause excitotoxic cell death [82, 85]. Seizure activity induced by mefloquine antagonism resulting from loss or dysregulation of SNr tonic firing could therefore give rise to a number of the side effects observed in clinical cases of mefloquine toxicity, including significant neuropsychiatric disturbances.

Effects of mefloquine exposure to cells of the SNr has been examined in some detail* in vitro* [94]. Mefloquine has been shown to cause hyperexcitation in primary dopaminergic neurons of the SNr, increasing pacemaker firing activity in a concentration dependent manner [94]. Significantly, this effect was observed at concentrations far lower than those found in the plasma of patients treated with mefloquine for malarial prophylaxis (0.3–10 mM compared to 3.8–23 mM in humans). This study also showed that this increased firing pattern enhanced GABA_A_-receptor mediated synaptic transmission by increasing intracellular calcium and inhibition of cholinesterase [94]. In a whole animal system, this could result in sustained binding of endogenous acetylcholine to its receptors, potentially causing some of the adverse neurobehavioural and cognitive effects reported in patients undergoing mefloquine treatment. Whilst this hypothesis does not account for all of the prodromal and acute neuropsychiatric symptoms associated with exposure to mefloquine, and further work is needed to evaluate the role of cholinergic stimulation in mefloquine toxicosis, it is compatible with a past description of mefloquine toxicity as a central anticholinergic syndrome [107] as well as known correlations between anticholinergic medications and impaired cognitive and motor function [108, 109].

Together these studies suggest that mefloquine blockade of K_ATP_ channels in the SNr could manifest as many of the abnormal behaviours, including heightened states of anxiety, aggression, antisocial or criminal behaviour, or seizures, widely associated with mefloquine toxicity, a hypothesis that could be investigated further in human patients experiencing adverse reactions to mefloquine prophylaxis or treatment [36, 37, 110, 111].

## 6. **K**_ATP_ Channels in Movement, Auditory, and Visual Pathways, Implications for a Role in Mefloquine Toxicosis

Further evidence exists to support a hypothesis of a role for K_ATP_ channels, and their blockade, in clinical presentation of movement, auditory, and visual disturbances associated with mefloquine toxicity. Neurons of the SNr interact with subcortical areas directly via the striato-nigral pathway and indirectly through the striato-pallido-subthalamic-nigral pathway, the former exerting a strong GABA-mediated inhibitory role on more posterior brain regions, including the cerebellum via connections in the pedunculopontine tegmental nucleus (Figure 2) [90, 112]. K_ATP_ channels are both pre- and postsynaptic in this pathway. GABAergic neurons of the striatum express K_ATP_ channels on their terminal axons, as well as postsynaptic channels being present on SNr neurons themselves [113]. In the nigropedunculopontine pathway, SNr neurons extend direct connections to pontine cerebellar structures, the superior colliculus, and the pedunculopontine tegmental nucleus [112], where they play a role in modulation of saccadic and pursuit eye movements in response to sensory and attention signals from the cortex, as well as balance and coordination. A dysregulation of inhibitory input to visual or auditory centres could therefore account for auditory and visual hallucinations frequently reported in cases of mefloquine psychosis [37, 114–117].

A link between neuronal activity in the SNr and K_ATP_ mefloquine blockade may also underlie some of the variation in presentation of adverse effects noted in travellers and patients using mefloquine for malarial prophylaxis or treatment. The SNr is a region of the brain slow to reach functional maturity [118] and shows sex specific differences in its activity [119]. It has been reported that children tolerate mefloquine treatment better than adults and male better than female patients [120]. These functional differences in the SNr between juveniles, adults, and the elderly, as well as between men and women, could account for some of the significant variation observed in adverse effects of mefloquine treatment and prophylaxis.

Genetic variance or mutation in the K_ATP_ channel subunit genes Kir6.2 and SUR1 could underlie some of the interpersonal variation observed in patients suffering from adverse effects of mefloquine exposure. Genetic variation in Kir6.2 and SUR1 subunits have been shown to cause both Type 1 and Type 2 diabetes, as well as epilepsy in humans [121–125]. As such, genetic variation in these genes might therefore predispose some individuals to more severe adverse events when taking quinoline derivatives, such as mefloquine, for malarial prophylaxis or treatment. Genetic screening for sequence variations in the Kir6.2 and SUR1 subunits in patients presenting with severe neuropsychiatric symptoms after mefloquine exposure could begin to define these connections more definitively and provide a better understanding as to the origin of the clinical variation presenting in cases of mefloquine toxicity.

## 7. **K**_ATP_ Channels, Connexins, and Intercellular Connections in Mefloquine Toxicity

The connection between mefloquine disruptions of interneuronal communication via blockade of connexins channels, a family of gap junction family proteins, is now well established [126]. Like K_ATP_ channels, connexins (Cx) play an important role in neuronal metabolism and homeostasis by controlling movement of ions, metabolites, and other molecules between adjacent cells of the CNS. Gap junctions establish electrical coupling by allowing intercellular exchange of ions and metabolic support by transport of ADP, glucose, glutamate, and glutathione, as well as movement of second messengers such as cyclic AMP. A wide variety of pharmacological agents have been shown to influence their activity [127] and dysfunction of connexin channel activity, or their blockade, has been implicated in a number of neuropsychiatric disorders common with mefloquine toxicity including suicide completion [128], vestibular dysfunction [129], and epilepsy [130].

There is a known functional connection between connexins and K_ATP_ channels in states of neuropsychiatric abnormality. It has been shown that K_ATP_ channels regulate the expression of Cx43 and Cx45 in the epileptic hippocampus [131]. Cx36 is widely expressed in cortical, subcortical, and limbic regions of the brain and has been implicated as a player in mefloquine toxicosis [132, 133]. Interestingly, Cx36 is also expressed by dopaminergic neurons of the SNr and GABAergic neurons of the ventral tegumental area [134, 135], again linking activity of connexins to those membrane effects of K_ATP_ channels in these regions. Both mefloquine and quinine have been shown to selectively block the activity of Cx36 and Cx50 channels* in vitro*, mefloquine with significantly higher potency than its parent molecule [126, 136, 137] and mefloquine inhibition of Cx36 in the inferior olive in humans has been shown to diminish motor learning skills [138].

A connection between mefloquine, connexins, and altered neurosensory function has also been identified. Mefloquine inhibits Cx26, dominant mutations of which have been shown to cause neurosensory deafness as well as attenuating increased membrane currents in primary cells expressing a dominant negative human Cx26 channel [139]. Cx26 is also expressed in neurons of the SNr [134]. The possibility of dual dysfunction of both K_ATP_ and connexin membrane channels (Figures 1(c) and 1(d)), giving rise to both intra- and intercellular changes in neuronal metabolism and activity, could therefore underlie some of the very severe neuropsychiatric events observed in cases of mefloquine toxicity.

Together, these studies suggest a synergistic role for connexins regulating intercellular excitability, and K_ATP_ channels regulating intracellular metabolism, in the pathogenesis of mefloquine toxicity via multiplex membrane channel blockade. Variation in the sensitivity of either or both membrane channels, due to age, genetic variation, interaction with other gap junction blockers, or channel antagonists, could explain the extreme variation in neuropsychiatric symptoms presenting in patients exposed to supposedly “safe” levels of mefloquine.

## 8. Conclusions

**Figure 3:**
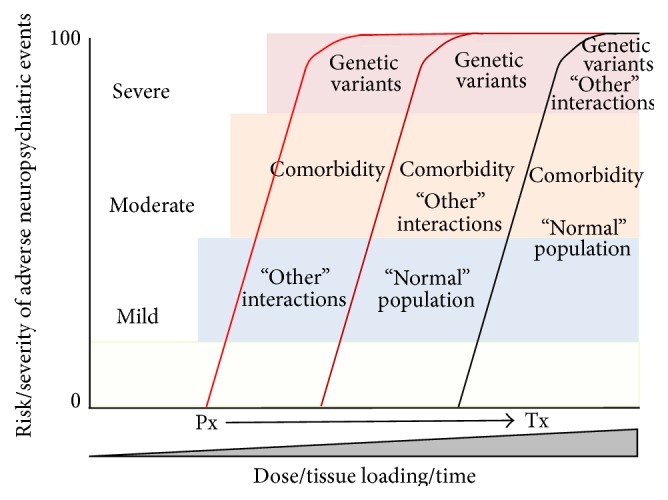
The hypothetical relationship between risk and severity of adverse neuropsychiatric reactions in patients exposed to prophylactic (Px) or treatment (Tx) doses of mefloquine and their relation to the presence of genetic variation, such as K_ATP_ channel subunit variants, CYP450, MDR1, and connexin allelic mutations, comorbidity with other neuropsychiatric disorders and “other” currently identified comorbid factors such as alcohol intake, low body mass index, age, immune suppression, and concurrent malarial disease. Within the “normal” population at low doses mild to moderate neuropsychiatric symptoms are common, with this proportion increasing with increasing doses at treatment. At low (Px) doses, individuals with predisposing conditions, such as pharmacogenetic predisposition, or comorbidity, will manifest more severe adverse reactions more quickly than the “normal” population. Individuals with additional compounding comorbid factors will present the most severe symptoms most quickly. Treatment doses will elicit the most severe symptoms, most quickly, in those with single or multiple predisposing conditions as well as in an increased proportion of the “normal” population exposed to this drug.

Significant evidence now exists for a primary role for membrane channel blockade in the presentation and severity of adverse neuropsychiatric reactions in patients exposed to mefloquine at normal prophylactic or treatment levels. How these complex cellular interactions manifest as neuroelectrophysiological and neurochemical changes, synaptic dysfunction, or neuronal cell death is still not clear but it seems likely that the delicate balance between excitation and inhibition caused by mefloquine exposure, both intra- and intercellularly, is likely to play a central role with connexins and K_ATP_ channels both implicated in this process. A diagrammatic representation of the complex interrelationship between risk and severity of adverse events, pharmacogenetics, comorbidity with other neuropsychiatric disorders, and dose/length of exposure is illustrated in Figure 3. Reduced activity or blockade of both of these membrane channels in the brain's central pacemaker and the substantia nigra pars reticulata, as well as increased sensitivity to mefloquine due to underlying allelic variation, could provide an overarching hypothesis bringing together many of the diverse neuropsychiatric events reported in cases of mefloquine toxicoses.

Further studies, including functional and structural imaging of deep brain regions in patients suffering from mefloquine toxicity and examination of electrophysiological changes in cells of the substantia with mutation or variation in both K_ATP_ and connexin channels on exposure to mefloquine, could begin to elucidate the delicate interplay between excitation and inhibition in cases of mefloquine toxicoses. A hypothesis of dysfunction of the central pacemaker, giving rise to mesolimbic dysregulation, could also provide novel treatment options for patients suffering from adverse reactions to mefloquine exposure in the future.
